# Communication of prostate cancer cells with bone cells via extracellular vesicle RNA; a potential mechanism of metastasis

**DOI:** 10.1038/s41388-018-0540-5

**Published:** 2018-10-23

**Authors:** C. Probert, T. Dottorini, A. Speakman, S. Hunt, T. Nafee, A. Fazeli, S. Wood, J. E. Brown, V. James

**Affiliations:** 1 School of Veterinary Medicine and Science, University of Nottingham, Nottingham, LE12 5RD UK; 2 Advanced Data Analysis Centre, University of Nottingham, Nottingham, LE12 5RD UK; 3 School of Clinical Dentistry, University of Sheffield, Claremont Crescent, Sheffield, S10 2TA UK; 4 Academic Unit of Reproductive and Developmental Medicine, University of Sheffield, Jessop Wing, Tree Root Walk, Sheffield, S10 2SF UK; 5 Department of Pathophysiology, Institute of Biomedicine and Translational Medicine, University of Tartu, Tartu, Estonia; 6 Department of Oncology and Metabolism, Western Park Hospital, University of Sheffield, Whitham Road, Sheffield, S10 2SJ UK

**Keywords:** Non-coding RNAs, Prostate cancer

## Abstract

The role of extracellular vesicles (EVs) as vehicles for cell-to-cell communication between a tumour and its environment is a relatively new concept. The hypothesis that EVs may be critical in co-opting tissues by tumours to generate distant metastatic niches is particularly pertinent to prostate cancer (PCa), where metastatic-tropism to bone predominates over other tissue types. The potential role of EVs as a means of communication between PCa cells and cells of the bone stroma such as osteoblasts, is yet to be fully explored. In this study, we demonstrate that PCa cell EVs both enhance osteoblast viability and produce a significantly more supportive growth environment for PCa cells when grown in co-culture with EV-treated osteoblasts (*p* < 0.005). Characterisation of the RNA cargo of EVs produced by the bone-metastatic PCa cell line PC3, highlights the EV-RNA cargo is significantly enriched in genes relating to cell surface signalling, cell–cell interaction, and protein translation (*p* < 0.01). Using novel techniques to track RNA, we demonstrate the delivery of a set of PCa-RNAs to osteoblast via PCa-EVs and show the effect on osteoblast endogenous transcript abundance. Taken together, by using proof-of-concept studies we demonstrate for the first time the contribution of the RNA element of the PCa EV cargo, providing evidence to support PCa EV communication via RNA molecules as a potential novel route to mediate bone metastasis. We propose targeting PCa EVs could offer a potentially important preventative therapy for men at risk of metastatic PCa.

## Introduction

Prostate cancer is the second leading cause of cancer-related death in men and the most commonly diagnosed male malignancy worldwide, with > 1.1 million cases recorded in 2012, which accounts for 15% of all new cancer cases in men [1]. In advanced PCa, 80% of men will have cancer that has metastasised to bone. At this stage, treatment options focus on palliative care and the 5-year survival rate for these patients is ~ 30% [2]. Why and how bone becomes the focus of PCa remains uncertain. In this study, we explore the RNA cargos of extracellular vesicles (EVs) secreted by PCa cells, to determine their effect on bone osteoblasts as a potential means of influencing PCa bone metastasis.

The concept of tumour-secreted factors, such as EVs, as a means of communication within the metastatic process is relatively new, but recent studies provide compelling evidence for further investigation [3–14]. For example, studies of TLR3^−/−^ knockout mice found the RNA cargo of melanoma EVs was capable of activating signalling pathways in lung epithelial cells, resulting in chemokine secretion and spontaneous lung metastasis, strongly supporting a role for cancer cell EVs in the metastatic process [9].

EVs are any type of lipid bilayer bound vesicle released into the extracellular space. Typically, EVs are classified based on their biogenesis and size, but they also differ in their molecular content, membrane composition, and specific functions. Classification by biogenesis and size depicts three core types of EV, exosomes that are the smallest in size and form from inward budding of the endosomal membrane (30–150 nm), and are thought to have a functional role in cell–cell communication [15, 16]. Microvesicles form from outward budding of the plasma membrane (> 50–1000 nm) and have classically been thought of as a means of evacuating waste/unwanted products from the cell [17, 18]. Finally, apoptotic bodies (50 nm–5 µm) are formed through the disassembly of other membrane-bound vesicles and released in the later stages of apoptosis [16]. EVs are known to carry a cargo of proteins and genetic material, of which a large variety of RNA species are found to be present including messenger RNAs (mRNA), microRNAs (miRNAs), transfer RNAs (tRNAs), small nucleolar RNAs (snoRNAs), small nuclear RNAs (snRNAs), mitochondrial-associated RNA, Piwi-interacting RNAs (piRNAs), vault RNAs, Y-RNAs, ribosomal RNAs, and long non-coding RNAs [19–24]. Identification of miRNAs in PCa vesicles, within the population classified as exosomes, has yielded some potential new prognostic markers [25–29], but the potential function of these EV-RNAs is yet to be determined.

In this study, we use in vitro models and novel RNA-tracking techniques to explore the RNA cargos of PCa EVs, which fall within the category of exosomes, to investigate the interaction between PCa cells and osteoblasts. Demonstrating a method of communication that has the potential to mediate PCa to bone metastasis.

## Results

### PCa EVs increase osteoblast viability creating an enhanced growth environment in vitro

**Fig. 1:**
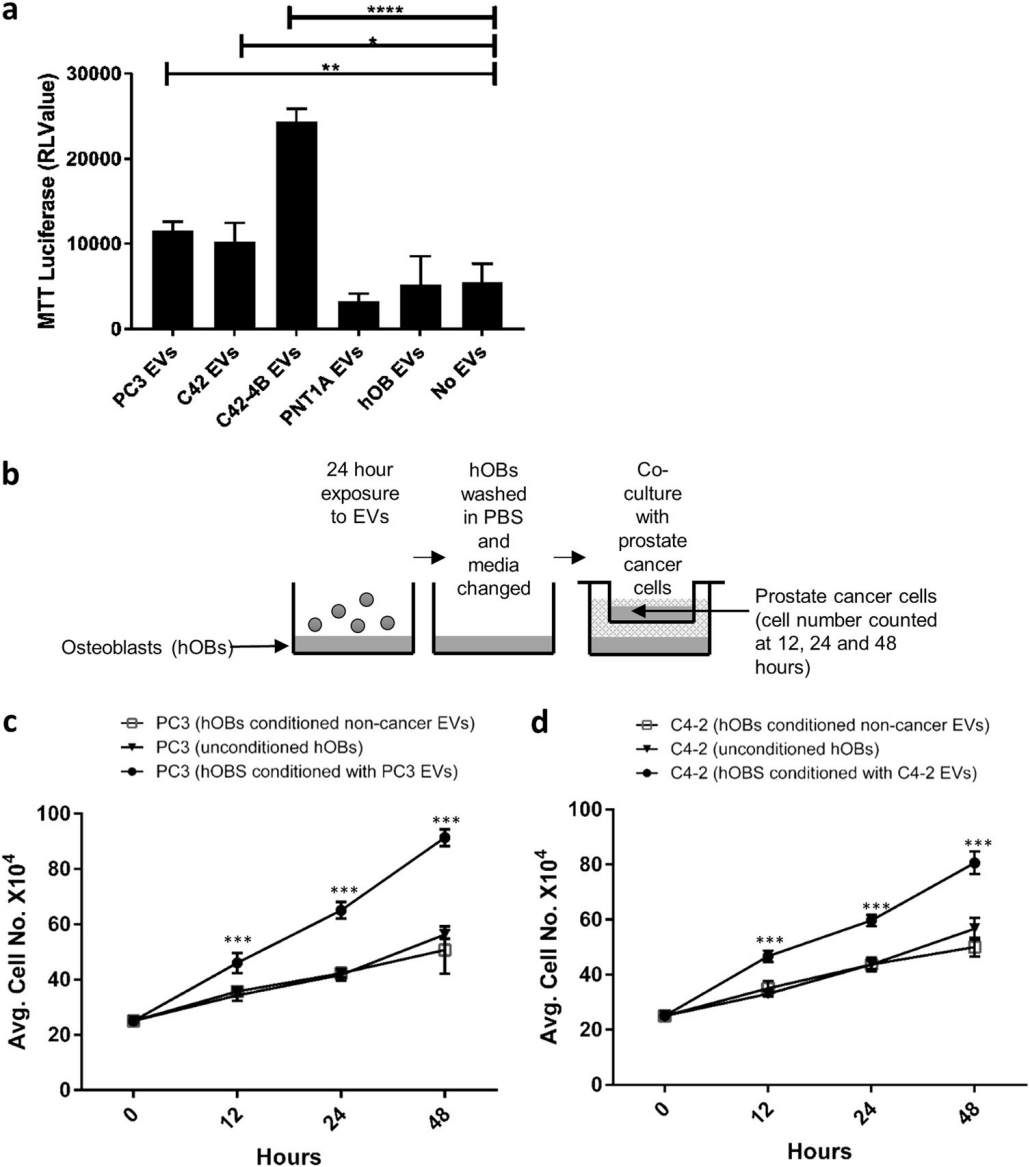
Extracellular vesicles isolated from prostate cancer cells with a bone-metastatic propensity educate osteoblasts to create an enhanced growth environment. **a** Osteoblasts (hOBs) were treated with extracellular vesicles (EVs) isolated from cultured prostate cancer cell lines PC3, C4-2, C42-B, PNT1A, the same hOB cell line or no EV control, cell viability was measured after 24 h using an MT luciferase assay. A significant increase in luciferase was detected in hOB cells treated with EVs from prostate cancer cell lines PC3, C4-2 and C42-4B (*p* = 0.004, *p* = 0.032, *p* = 0.0001, respectively) (*n* = 3). **b** To determine whether the exposure of osteoblasts to EVs creates and environment supportive of prostate cancer cell growth, osteoblasts (hOBs) were pre-treated by incubating cells for 24 h with extracellular vesicles (EVs) isolated from cultured prostate cancer cell lines PC3 and C4-2, HEK-293 as a non-cancer control, or with PBS only. After 24-hours, hOBs were washed in PBS and co-cultured with the same prostate cancer cell line used to isolate the EVs (during co-culturing the prostate cancer cells were grown on cell culture inserts to prevent cell-to-cell contact. Cell number was measured at 12, 24 and 48 h (in triplicate) and changes in growth rates compared across the different EV treatments. **c** Pre-treatment of hOBs with PC3-EVs for 24 h as described above, resulted in a significant increase in PC3 cell number during co-incubation across all time points, compared with hOBs incubated with non-cancer HEK-293 EVs or PBS. **d** As seen in **c**, pre-treatment with C4-2 EVs as described above, resulted in a significant increase in cell number compared to HEK-293 EVs and PBS alone. **a**, **c**, **d** *P* values determined using a two-way ANOVA and Bonferroni’s multiple comparison test **b** *P* values determined using one-way ANOVA and Holms-Sidak correction, error bars represent standard deviation *n* = 3. *p ≤ 0.05, **p ≤ 0.01, ***p ≤ 0.001, ns not significant

To investigate if PCa EVs have the potential to affect cell types which may reside within a potential bone-metastatic niche, we first sought to determine whether EVs isolated from PCa cells PC3 (which has a propensity to metastasis to bone in vivo) C4-2, (which shows occasional metastasise to bone in vivo [30]), and C4-2-4B (a bone-metastatic lineage of C4-2 [30]) could induce a change in osteoblast viability. An immortalised osteoblast cell line (hOB) was exposed to one dose of EVs isolated from PC3, C4-2, C4-2-4B PCa cells or PNT1A (a non-malignant immortalised prostate epithelial cell line) or a separate population of hOB cells, treatment with media containing no EVs was used as a negative control. EVs were characterised as being within the category of exosomes as determined by Brownian motion (Zetaview, ParticleMetrix) and immunoblot analysis (Supplementary Figures 1 and 2). Treatment with all PCa EVs (PC3, C4-2, C4-2-4B) resulted in a significant increase in hOB viability 24 h after treatment (*p* = 0.004, *p* = 0.032, *p* = 0.0001 respectively) (Fig. 1a). Similar results were found when using a second osteoblast cell line hFOb1.19 (Supplementary Figure 3).

To determine whether the changes in osteoblast viability, induced by PCa EVs, result in a supportive growth environment for subsequently arriving PCa cells (as a rudimentary model of early metastasis). Osteoblasts (hOBs) were incubated with EVs isolated from PC3, C4-2 or the non-PCa cell line Hek293 for 24 h, prior to the addition of PCa cells to form a co-culture system (illustrated in Fig. 1b). The number of the co-cultured PCa cells was measured over 48 h. The co-culture of PC3 cells, with hOB cells that had been pre-treated with PC3-derived EVs, resulted in a significant increase in PC3 cell number when compared with PC3 cells co-cultured with hOB cells pre-treated with EVs isolated from the non-prostate HEK293 cells (12 h *p* = 0.0015; 24 and 48 h *p* < 0.0001), or no pre-treatment (12 h *p* = 0.0004; 24 and 48 h *p* < 0.0001) (Fig. 1c). The growth of C4-2 cells within the co-culture system was similarly influenced by pre-treatment of hOB cells with C4-2-derived EVs (compared with no treatment 12, 24 and 48 h *p* = 0.0004, and Hek293 EVs 12, 24 and 48 h *p* < 0.0001) (Fig. 1d).

Taken together, these data indicate PCa EVs alter hOB viability. Moreover, the EV-mediated changes that result from pre-treatment of hOB cells result in increased titres of later arriving PCa cells, an effect not replicated by EVs of non-prostate cell types. Therefore, demonstrating PCa EVs deliver a pro-tumour cell signal. As we demonstrated PCa EVs were able to affect the viability of osteoblasts, a major cell type affected in PCa bone metastasis, we choose to determine the role of the EV cargo. As other studies have identified some protein components of cancer EV cargos, we choose to ascertain the potential contribution of the RNA cargo of PCa EVs.

### Characterisation of PCa EV-miRNA

**Fig. 2:**
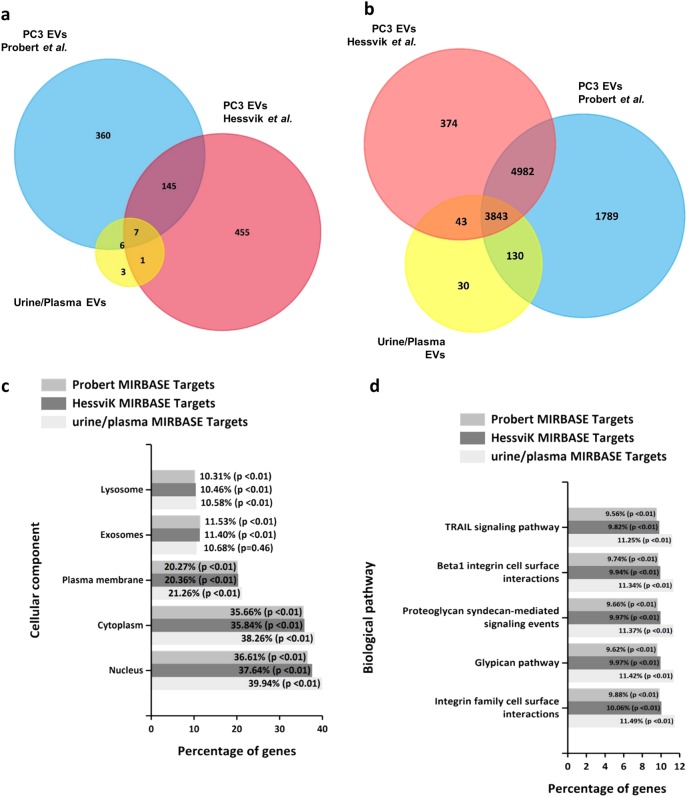
Analysis of the potential function of PC3 EV-miRNAs and a comparison of mapped microRNA between publically available studies of the PC3 cell line and prostate cancer patient urine and plasma EVs. Data from our study (Probert 2017), Hessvik et al., a similar study of PC3-EVs [31] and published findings of miRNAs with prognostic relevance identified in EVs isolated from patient urine and plasma [25–27, 29] were mapped to miRBase to create a data set for the comparative analysis described **a**–**d**. **a** Venn diagram to show microRNAs shared between the studies. In all, 40.3% of miRNAs identified in our study were also reported to be present in PC3 cell line isolated EVs studied by Hessvik et al. [31]. In comparison, 76.5% of miRNAs detected in urine and plasma EVs isolated from patients and reported to have prognostic potential, were also present in our study. **b** Venn diagram to show the mRNA targets of the EV-miRNA cargos across the described studies [25–27, 29, 31]. **c** Comparative cellular component analysis of the mapped microRNAs across the studies demonstrate similar percentages of target genes in the same cellular compartments irrespective of the study/origin of the EVs (cell line or patient sample origin). **d** The same is the case when comparative biological pathway analysis is applied. The characterised major biological pathways potentially targeted by the EV-miRNA cargos represent cell surface/membrane signalling and interaction mechanisms. Venn diagrams and comparative analysis conducted using FUNRICH V3 [45]

To further explore the RNA cargo of PCa EVs, RNAseq was used to identify the miRNA and mRNA cargo of EVs isolated from the bone-metastatic PC3 PCa cell line. Sequencing reads were mapped using miRBase to identify miRNAs and to allow a comparison to the previous study of the miRNA content of PC3 exosomes [31] (the only full data set publically available GSE35813 at the time of study). A comparison of the two studies identified 40.3% of the miRNAs identified in our study were also reported by Hessvik et al. [31] (Fig. 2a). When comparing this with reports of miRNAs with prognostic significance (associated with progression to metastasis and/or detection of high/intermediate risk PCa) detected in EVs isolated from PCa patient plasma and urine, 76.5% of the these miRNAs were identified in our study compared with 47.1% in the Hessvik et al. study [25–27, 29, 31] (Fig. 2a) (details of the in common miRNAs are reported in Supplementary Table 1). Variations in the miRNA cargos of vesicles may be a representation of the biological heterogeneity of EVs and the influence of different stimuli encountered during the culture process [32]. An additional source of differences may also occur through the process of isolating vesicles via size exclusion and ultracentrifugation techniques, as well as the use of RNAseq versus array-based technologies to profile the miRNAs [33]. However, these comparisons demonstrate a core of miRNAs can be detected in PCa EVs across studies.

In addition to identifying the miRNA types, determining the targets of those miRNAs demonstrates that between our study of PC3-EVs and Hessvik et al., 82.1% and 96.6% of predicted gene targets are shared, respectively (Fig. 2b). Extending the comparison of miRNA gene targets with the studies of patient plasma and urine, 94.98% of genes targeted by the miRNAs in those studies are in common with our study (Fig. 2b). These data indicate an overlap in genes targeted by EV-miRNAs despite some difference in the miRNA composition of those EVs, this is further supported by comparative cell component and biological pathway analysis. Cell component analysis identified a significant enrichment of genes within different cell compartments, targeted by the all miRNAs identified in our study and all miRNAs identified in the studies used in the previous comparison [25–27, 29, 31]. The top five cell compartments showing enrichment of gene targets were as follows nucleus (*p* < 0.01), cytoplasm (*p* < 0.01), plasma membrane (*p* < 0.01), exosomes (*p* < 0.01 except for studies of urine and plasma EVs), and lysosomes (*p* < 0.01) (Fig. 2c). All studies showed a similar percentage of target genes across these five components. This is further exemplified through comparative biological pathway analysis, the top five pathways showing a significant enrichment of target genes were Integrin family cell surface interactions, Glypican pathway, Proteoglycan syndecan-mediated signalling events, Beta1 integrin cell surface interactions, and TRAIL signalling (all *p* < 0.01) (Fig. 2d). All five pathways correspond to cell surface signalling and cell interaction mechanisms.

Following the characterisation of the miRNA cargo, labelling of nascent RNA with 5-Ethynyl Uridine (5EU) in PC3, non-malignant PNT1A and hOB cells was used to track RNA originated from EVs, to determine whether miRNAs transferred from PC3-EVs could be detected in recipient osteoblasts.

**Fig. 3:**
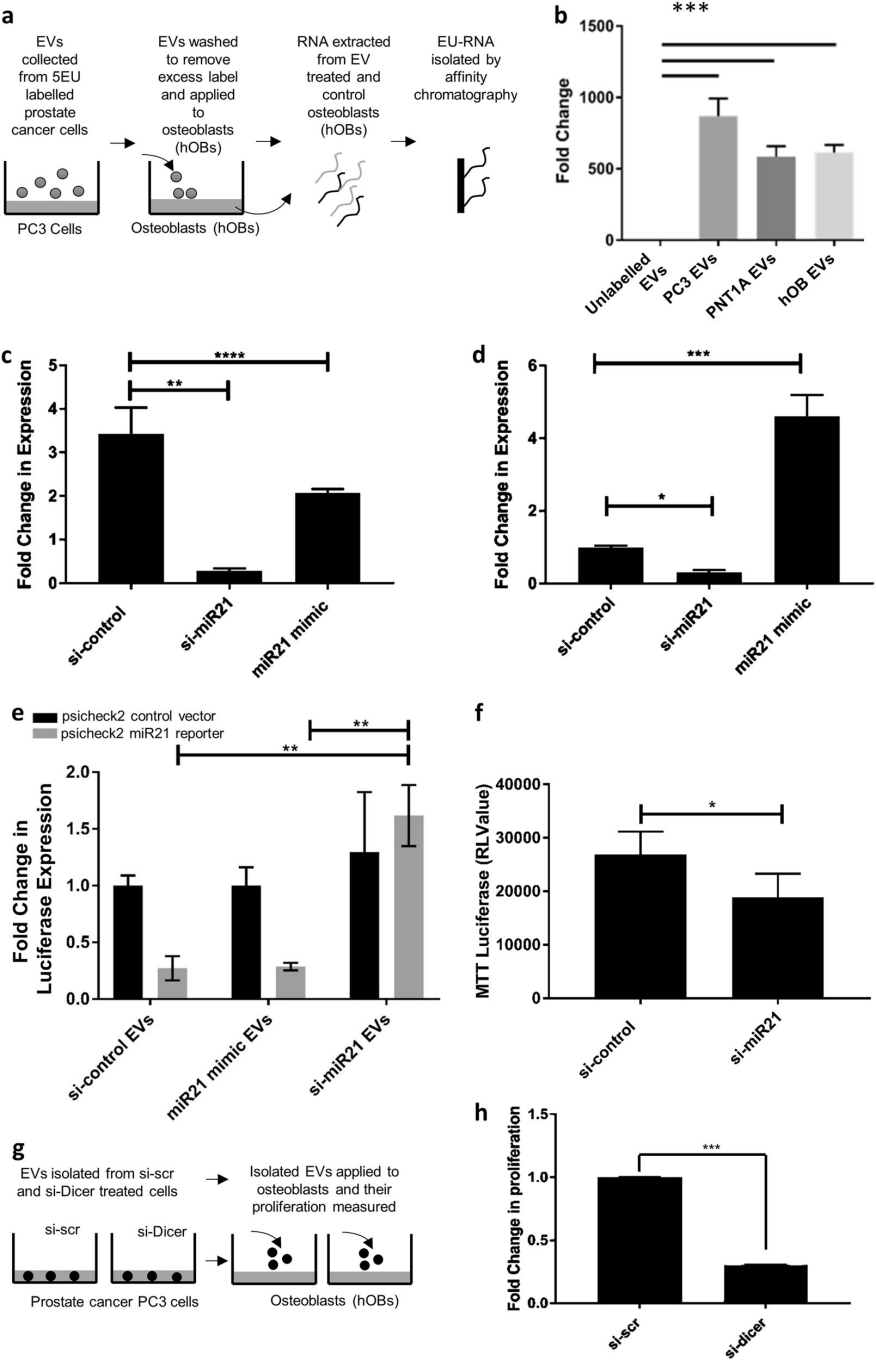
Detection of labelled microRNA-21 (miR21) originating from bone-metastatic prostate cancer cell lines in recipient osteoblasts and functional effects of EV-miRNAs. **a**–**b** PNT1A (normal prostate), PC3 (prostate cancer) or hOB (osteoblast) cells were grown in the presence of 5EU to label nascent RNA transcripts. Post-labelling EVs produced from these cell lines were isolated and applied to hOBs cells grown under standard conditions (no EU label). After 48 h, the EV-treated hOB cells were lysed and the total RNA extracted, from the pool of total RNA 5EU-labelled RNA was precipitated and the presence of labelled miR21 determined by qPCR**. c** Quantification of mature miR21 abundance in PC3 cells 48 hours post transfection with siRNAs targeting miR21 hairpins, miR21 mimics or a non-targeting siRNA control (*n* = 3). **d** Quantification of mature miR21 abundance in EVs isolated from PC3 cells 48 hours post transfection with siRNAs targeting miR21 hairpins, miR21 mimics or a non-targeting siRNA control (*n* = 3). **e** To determine whether the EV-miR21 can elicit a functional response, psiCHECK2 luciferase reporter assays containing miR21-binding sites were transfected into osteoblasts 48 h prior to treatment with EVs isolated from PC3 cells. Treatment with PC3-EVs, modified to deplete miR21 from the EV cargo, resulted in a significant increase in the expression of the luciferase reporter (*p* = 0.00002) (*n* = 3). **f** Exposure of hOB cells to miR21-depleted PC3-EVs also results in a reduction in hOB cell viability (*p* = 0.0134). **g–h** To determine the potential contribution of RNAs to the modification of the osteoblast phenotype by EVs, PC3 cells were transfected with siRNA against dicer (si-dicer) or a non-targeting siRNA (si-scr) control (Supplementary Figure 4). After 48 h post of transfection EVs were isolated from the PC3 cells and transferred to hOB (osteoblast) cells growing under normal conditions. The cell titre of hOB cells was determined 24 h after treatment with EVs isolated from PC3 cells treated with either si-scr or si-dicer (*n* = 3 biological replicates). **h** Treatment with EVs isolated from si-dicer-treated PC3 cells resulted in a significant reduction in hOB cell titre (*p* < 0.0001), indicating the involvement of miRNAs and/or the biogenesis pathway in altering the behaviour of EV-recipient cells. *t* test with Holm–Sidak correction error bars represent standard deviation *n* = 3. *p ≤ 0.05, **p ≤ 0.01, ***p ≤ 0.001

Affinity capture was used to isolate the 5EU-labelled RNA present in EV-recipient hOB cells, and the captured 5EU RNA was subsequently probed for the presence of miR21 by qPCR (Fig. 3a). MiR21 was chosen for our study as its transfer between ovarian stromal cell types has previously been demonstrated [34]. Moreover, miR21 is known to be expressed in our cell lines and has been identified as a prognostic factor in the urine and plasma of PCa patients [27, 29]. Labelled 5EU-miR21 was detected in hOB cells following exposure to EVs isolated from all 5EU-treated cell lines (PC3, PNT1A and hOB) (*p* < 0.001 when compared with a background control), with the greatest abundance detected following treatment with PC3-EVs (Fig. 3b). To determine the potential functional effect of EV-miRNAs, PC3 cells were transfected with either a miR21 mimic to increase miR21 levels or an siRNA to deplete pre-miR21 hairpins to decrease the abundance of mature miR21 within the cells prior to the isolation of EVs, or a non-targeting small RNA control (si-control) (Fig. 3c). The abundance of miR21 was quantified in the isolated PC3-EVs (Fig. 3d), and the EVs subsequently applied to hOB cells transfected with a luciferase reporter containing miR21-binding sites within the 3’UTR. Luciferase levels were determined 24 h after treatment with EVs. Exposure of hOB cells to PC3-EVs depleted of mature miR21 (si-miR21 EVs) resulted in a significant increase in luciferase translation (*p* = 0.00002) (Fig. 3e). Furthermore, exposure of hOBs to miR21-depleted PC3-EVs resulted in a reduction in cell viability (*p* = 0.013) (Fig. 3f).

To determine the effect of PCa miRNAs on osteoblasts more generally, the miRNA biogenesis pathway of PCa PC3 cells was abrogated using Dicer RNAi (Supplementary Figure 4). Osteoblasts treated with EVs isolated from Dicer-depleted PC3 cells resulted in a significant reduction in osteoblast number (*p* < 0.0001) (Fig. 3g, h), indicating an RNA-dependent effect on osteoblast viability.

Using miR21 as an example, we have been able to confirm the transfer of PCa cell-derived miR21 transcripts to osteoblasts via PCa EVs. Furthermore, depleting the mature miR21 content of the PCa EVs results in loss of miR21-specific miRNA-mediated translational repression. This finding is in keeping with the work of Sanchez et al., (2016), who identified miR21 together with miR100 as the most abundant vesicle miRNAs in PCa. Furthermore, the authors found miR21 contributes to pre-metastatic niche preparation when introduced to stromal cells [35]. Although we cannot confirm if these data are unique to miR21, this finding together with the effect of Dicer-depletion on osteoblast number (Fig. 3g) support a functional role for PCa EV-miRNAs.

### Characterisation of PCa EV-mRNA

**Fig. 4:**
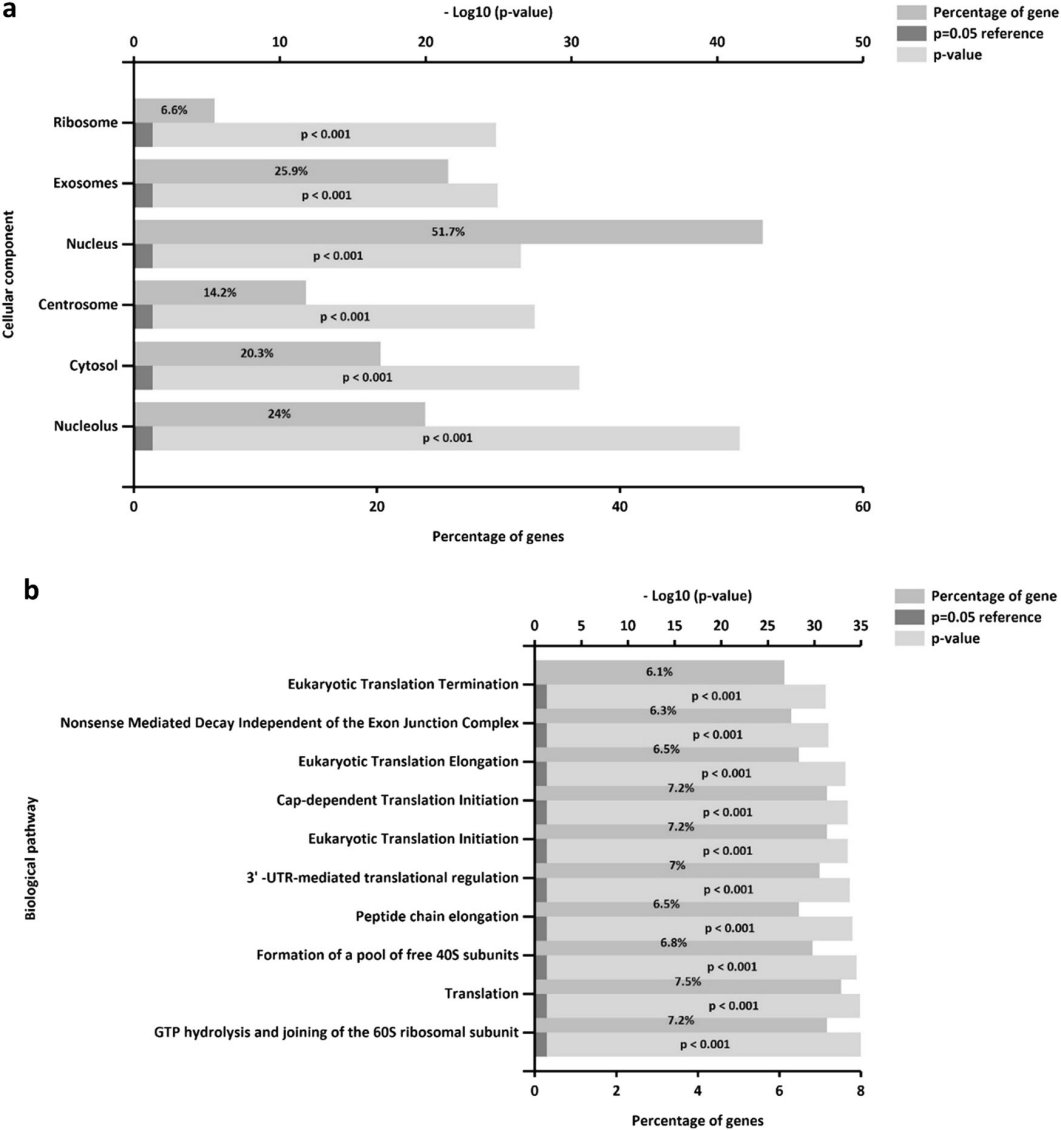
Analysis of messenger RNA (mRNA) transcripts present in PC3-EVs identifies an enrichment of genes involved in polymerase III transcription. **a** Cell component analysis demonstrates a significant enrichment of genes involved in nuclear functions (Nucleus, Nucleolus and Centrosome) and cytoplasmic functions involved in translation (ribosomes) and endosomal vesicle formation (exosomes). **b** Biological pathway analysis indicates a significant enrichment of process involved in protein translation (top 10 shown, full analysis detailed in Supplementary Table 2)

In addition to miRNAs, 572 mRNA transcripts were identified by RNAseq analysis of PC3-EVs. Exploration of the potential functions of this population by cellular compartment analysis indicates an enrichment of genes involved in both nuclear and cytoplasmic functions (Fig. 4a). Perhaps more informative, biological pathway analysis shows a predominance of genes involved in protein translation, the top 10 pathways are illustrated in Fig. 4b (full list detailed in Supplementary Table 2).

**Fig. 5:**
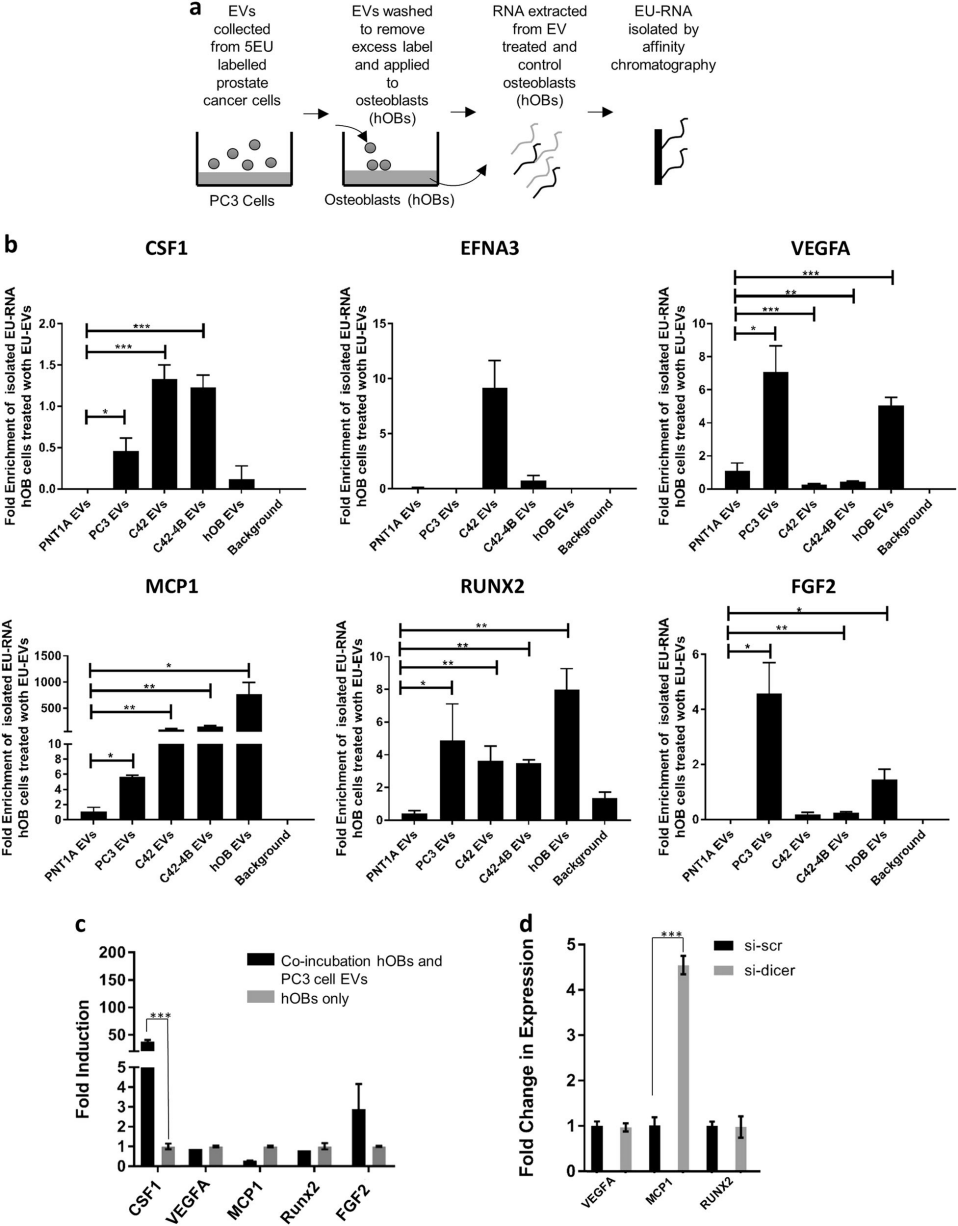
Detection of labelled mRNAs originating from bone-metastatic prostate cancer cell lines in recipient osteoblasts and the contribution to overall transcript abundance in recipient osteoblasts. **a** PNT1A (normal prostate), PC3, C4-2, C4-2-4B (prostate cancer) or hOB (osteoblast) cells were grown in the presence of 5EU to label nascent RNA transcripts. Post-labelling EVs produced from these cell lines were isolated and applied to hOBs cells grown under standard conditions (no EU label). After 48 h, the EV-treated hOB cells were lysed and the total RNA extracted, from the pool of total RNA EU-labelled RNA was precipitated and the presence of labelled CSF-1, VEGFA, MCP1, Runx2 and FGF2 quantified. **b** All EU-labelled transcripts were detected at significantly higher levels in hOB cells treated with EVs isolated from EU-labelled PC3 cells compared with EU-labelled PNT1A cells (CSF-1 *p* = 0.0395, VEGFA *p* = 0.0134, MCP1 *p* = 0.0086, Runx2 *p* = 0.0168, FGF2 *p* = 0.0284) (*n* = 3). Similar treatment with prostate cancer C4-2 and C42-B4 EVs, resulted in detection of increased 5EU-labelled CSF-1, MCP1 and RUNX2, but not VEGFA or FGF2 as seen with PC3. (*p* = 0.001, *p* = 0.001, *p* = 0.001 and *p* < 0.0001, *p* = 0.001, *p* = 0.002, respectively) (*n* = 2). **c** Analysis of total RNA extracted from hOBs after treatment with labelled PC3-EVs in **a** demonstrated a significant increase in abundance of CSF-1 and a smaller increase in FGF2 (similar results were obtained following treatment with C4-2 EVs Supplementary Figure 6). **d** To determine how the miRNA element of the EV cargo may influence the subsequent processing of the mRNA cargo component in the recipient cell, PC3 cells were transfected with siRNA against dicer (si-dicer) or a non-targeting siRNA (si-scr) control. 48 h post transfection EVs were isolated from the PC3 cells and transferred to hOB (osteoblast) cells growing under normal conditions. The abundance of VEGFA, MCP1 and Runx2, shown previously to have no altered abundance within the osteoblast transcriptome **a–b**, were quantified. A significant increase in the expression of MCP1 (*p*<0.0001) was determined, but no change in VEGFA and Runx2 could be detected (*n* = 3). Western blot confirmation of Dicer knockdown and controls for the siRNA loading into EVs is included in Supplementary Figure 4. *t* test with Holm–Sidak correction error bars represent standard deviation *n* = 3. *p ≤ 0.05, **p ≤ 0.01, ***p ≤ 0.001

Similar to the study of EV-miRNAs, we sought to determine whether the mRNA cargo of PCa EVs could also be detected in the recipient osteoblasts (Fig. 5a). We analysed the abundance of five genes that, based on our RNAseq data, were known to be expressed in the EVs of PCa cell lines (Supplementary Figure 5). Furthermore, this subset of genes have key roles in osteoblast function and consist of colony-stimulating factor 1 (CSF-1*)* a factor present on the osteoblast cell surface and secreted by osteoblasts to mediate osteoclast formation [36], Ephrin A3 (EFNA3*)* required for osteoblast cell–cell interaction and osteoblastic bone formation [37], vascular endothelial growth factor A (VEGFA*)* osteoblasts are stimulated to produce VEGFA in response to bone morphogenetic proteins to couple angiogenesis and bone formation processes [38], C–C motif chemokine ligand 2 (MCP1) produced by osteoblasts and hypothesised to be involved in the recruitment of osteoclast precursors and an activator of NF-KappaB ligand induced osteoclastogenesis [39], Runt-related transcription factor 2 (RUNX2) the constitutive expression of which is required to maintain the mature osteoblast phenotype [40], and fibroblast growth factor 2 (FGF2) expressed by osteoblasts and an important regulator of bone formation [41].

Osteoblasts were treated with EVs isolated from 5EU-labelled cells and the abundance of detectable 5EU-labelled mRNA transcripts within the treated hOB cells determined by qPCR (Fig. 5a, b). When compared with hOBs treated with EVs isolated from 5EU-labelled normal prostate epithelial cells (PNT1A), treatment with PC3-EVs resulted in a significant increase in 5EU-labelled RNAs transcripts for CSF-1 (*p* = 0.033), VEGFA (*p* = 0.013), MCP1 (*p* = 0.009), RUNX2 (*p* = 0.016) and FGF2 (*p* = 0.028) (Fig. 5b). Similarly, treatment with PCa C4-2 and C42-B4 EVs, resulted in detection of increased 5EU-labelled CSF-1, MCP1 and RUNX2, but not VEGFA or FGF2 as seen with PC3. (*p* = 0.001, *p* = 0.001, *p* = 0.001 and *p* < 0.0001, *p* = 0.001, *p* = 0.002, respectively). In the case of EFNA3, 5EU-labelled transcripts were not detected following treatment with PC3 or hOB EVs, but were detected after exposure to EVs from the less bone-metastatic C4-2 cell line (Fig. 5b). Treatment of osteoblasts (hOB cells) with EVs isolated from a separate population of 5EU-labelled hOB cells, also resulted in the detection of labelled CSF-1, VEGFA, MCP1, RUNX2 and FGF2 transcripts within the EV-recipient hOB cells (Fig. 5b).

As equal doses of EVs (based on total protein and EV characterisation) were applied to osteoblasts, differences in the osteoblast-transferred RNA cargos between EVs originating from different cell types suggest PCa EV-RNA cargos are likely tailored to promote changes in osteoblasts and/or other cells of the tumour microenvironment that would be beneficial to the tumour, as seen in the previous co-culture assays (Fig. 1c, d). For example, EFNA3 mRNA transcripts, whereas a detectable component of the PC3 EV cargo, appear not to be transferred at high abundance by EVs resulting from either bone-metastatic cell lines PC3 and C4-2-4B, but are transferred by C4-2, Further support for the presence of highly tailored PCa EV-RNA cargos is provided by our comparative analysis, which determined a significant enrichment of genes involved in key gene expression pathways (Fig. 4). However, the changes promoted by such cargos are yet to be modelled in vivo for PCa.

The transfer of mRNAs by EVs raises the question of the contribution they make to the overall transcriptome of the recipient cell. Do they simply increase the overall abundance or are they subject to regulation? By determining the abundance of CSF-1, VEGFA, MCP1, Runx2 and FGF in hOB cells exposed to PC3-EVs, we found the abundance of CSF-1 was significantly increased (*p* < 0.001). The level of FGF2 was also increased albeit significance was not reached. There was no significant change in the abundance of VEGFA, MCP1 or Runx2 (Fig. 5c). Similar results were found when using EVs isolated from PCa C4-2 cells (Supplementary Figure 5). The lack of increased abundance of MCP1, VEGFA and Runx2 was particularly marked, as compared with CSF-1 the abundance of these transferred mRNAs was between 4–6-fold higher (Fig. 5b). Therefore, other mechanisms of regulation must be active to control the overall levels of these transcripts within the osteoblast, signifying the possibility that the recipient osteoblast is only subject to the influence of a proportion of the RNAs present within the EV. Although it highly likely the source of RNA regulation is predominantly from the EV-recipient cell, we identified that 3.5% of the mRNAs identified within PC3 vesicles by RNAseq are targets of the vesicle miRNA cargo (Supplementary Figure 7). Therefore, we questioned if the miRNA cargo of the EVs influences the overall expression of MCP1, VEGFA and Runx2 in the recipient osteoblast. Using EVs isolated from PC3 cells treated with Dicer RNAi, we reanalysed the abundance of VEGFA, MCP1 and Runx2, finding only a significant increase in the abundance of MCP1 (*p* < 0.0001) (Fig. 5d). Therefore, indicating EV-miRNA-mediated negative regulation, whereas applicable to MCP1, is not a general mechanism of regulating mRNAs carried within the EV cargo.

The presence of mechanisms to regulate the EV cargo in the recipient cell, to fine tune the messages received and responded too, may prove to be vital in advancing our understanding of how PCa cells hijack the normal physiological processes mediated by EVs in the bone (reviewed ref. [42]), and importantly how therapeutic agents can be targeted to disrupt only the non-physiological/cancer-specific processes mediated by EVs.

## Discussion

The role of EVs as vehicles for cell-to-cell communication between a tumour and its environment is a relatively new concept, with only limited study of this potential mechanism in PCa. Our data are the first to demonstrate the transfer of RNA molecules from PCa cells to recipient osteoblasts and the importance of PCa EVs in changing osteoblast behaviour to support PCa cell proliferation. These data are in keeping with similar studies of melanoma EVs, where EV-RNA cargos were associated with mediating spontaneous lung metastasis [9]. The characterisation of the RNA content of PCa EV delineates the targeting of pathways important for osteoblast function and interaction with the surrounding microenvironment, both of which are in keeping with the changes in osteoblast viability observed following treatment with PCa cell EVs. Furthermore, we provide evidence of the function of transferred miRNAs in EV-recipient osteoblasts, and demonstrate the impact of RNA regulation on the retention of EV-RNA molecules, highlighting a mechanism potentially enabling the recipient cell to select, which messages within the EV communication system to ‘listen’ too.

This proof-of-principle study provides the critical evidence needed to warrant investigation of this complex inter-cell communication system in the context of in vivo models of PCa, particularly the potential of EVs to act in promoting metastasis. We propose further investigation using in vivo models are required to determine whether this form of communication acts as an initial or late event within the metastatic process. However, at the current time the tools required to delineate the functional role of EV-RNAs within a complex in vivo model of prostate bone metastasis are not yet available. The techniques successfully used in this study provided a basis for the development of future in vivo reagents to track the biodistribution of EV-RNA cargos combined with functional readouts of disease progression such as the extent of metastasis. Overall, these data provide an exciting step forwards in our understanding of how PCa cells use the EV-RNA cargo to communicate with osteoblasts and highlights the potential importance of the EV-RNA cargo as a mediator of PCa progression.

## Materials and methods

### Cell lines and culture conditions

PCa PC3, Hek293 and hfOb1.19 cells were obtained from ECACC (Culture Collections, Salisbury, UK) and cultured in Dulbecco’s modified medium (#D5796, Sigma, Dorset, UK) supplemented with 10% fetal bovine serum (#B9433, Sigma), 50 U ml^−1^ penicillin and 50 µg ml^−1^ streptomycin (#15140122, Gibco, Loughborough, UK). C4-2 cells were provided in collaboration by Dr Nigel Mongan (University of Nottingham) and C4-2-4B by Dr Penny Ottewell (University of Sheffield), cells were cultured as described [43]. The hOB osteoblast cell line was derived from adult femoral head trabecular bone [44] and was provided in collaboration with Dr Susan Anderson (University of Nottingham), hOB cells were cultured as described [19].

### Isolation and characterisation of EVs

Collection of EVs was carried out by seeding 1 × 10^6^ cells per 10 cm^2^ dish and incubating overnight. Following the overnight incubation, cells were washed in phosphate-buffered saline (PBS) and fresh media containing exosome-free fetal bovine serum (#A2720801, Gibco) applied. Cells were incubated for 48 h under standard conditions. After 48 h, the media was collected and vesicles isolated using the exoEasy Maxi kit (#76064, Qiagen, Manchester, UK) and gravity flow chromatography (#qEV, Izon Science, Oxford, UK) following the manufacturers’ protocols to isolate vesicles of less than 200 nm and within the range considered to represent exosomes. EVs were characterised by electrophoresis and Brownian motion analysis using laser scattering microscopy (ZetaView, ParticleMetrix, Meerbusch, Germany). Total protein was determined using the Qubit Protein Assay Kit (#Q33211, ThermoFisher, Loughborough, UK) and the Qubit 3.0 instrument (ThermoFisher). EV protein markers were assessed by immunoblot as subsequently described in the Protein extraction and immunoblots section.

### Co-incubation and EV treatment of hOB cells

Co-incubation experiments were performed by seeding 3 × 10^5^ PCa cells into 0.4 µm tissue culture inserts (#657641, Greiner Bio-one, Stonehouse, UK) in 3 ml media and 2 × 10^5^ hOB cells per well in six-well plates. Cells were incubated for 24 h prior to the transfer of inserts into six-well plates containing hOB cells. The two cell types were co-incubated for a further 48 h prior to analysis.

To treat cells with EVs, recipient cells (hOBs) were seeded into six-well plates at 2 × 10^5^ cells per well. Following an overnight incubation, the media was replaced with media containing exosome-free fetal bovine serum (#A2720801, Gibco) and cells were grown for 24 h prior to the application of isolated EVs.

### RNA labelling and subsequent labelled RNA isolation from hOB cells

5EU labelling of nascent RNAs was achieved by the addition of 5EU to the cell culture media at a final concentration of 0.4 mM for 24 h. To isolate labelled 5EU mRNAs from recipient cells, the Click-iT Nascent RNA Capture Kit (#C10365, ThermoFisher) was used, following the manufacturers recommended protocol. Unlabelled controls were used to determine the background level of RNA recovery of the precipitation step.

### RNAi transfection

Depletion of Dicer was achieved using Mission siRNAs (Dicer: SASI_Hs01_00160748, si-scr: SIC001, Sigma), modulation of miR21 was achieved using a custom-designed siRNA or Mission miR21 mimic (HMI0371/2, Sigma, Salisbury, UK). Cells were seeded at 3 × 10^4^ in 24-well plates and incubated overnight at 37 °C at 5%CO_2_. Cells were subsequently transfected with 20 nM siRNA using 1 µl Lipofectamine RNAiMAX reagent (#13778150, ThermoFisher), or 90 nM mimic miR21 and 4.5 µl Lipofectamine RNAiMAX following the manufactures recommended protocol for forward transfection.

### Luciferase reporter assays

Reporter constructs were created by cloning the miR21 seed sequences into the 3’-UTR of the psiCHECK2 dual luciferase reporter plasmid (#C8021, Promega, Southampton, UK). hOB cells were seeded into a 96-well plate at a density of 8 × 10^3^ cells per well, and subsequently transfected with 20 ng of psiCHECK2 plasmid DNA using 0.3 µl of Fugene HD transfection reagent (#E2311, Promega) following the manufactures recommended protocol. Forty-eight hours post transfection, cells were washed three times in PBS prior to the application of EVs in exosome-free media. Cells were lysed 72 h post- transfection using 100 µl Passive Lysis buffer (#E1941, Promega) and the luciferase luminescence read using the Dual-Glo Luciferase Assay System (#E2920, Promega).

### Measurement of cell viability

Assessment of cell viability was made by measuring the cell titre at the time points detailed. Cell titre was determined using both manual haemocytometer counts (without trypan blue) and the CellTiter-Glo assay system (#G7570, Promega) following the manufacturer’s protocol and luciferase readings measured using a GloMax-Multi Jr Single-Tube Multimode Reader (E6076, Promega) using the Cell-titre-Glo default settings. Viability was determined using RealTime Glo MT Viability assay system (#G9711, Promega), following all steps of the manufacturer’s protocol and readings taken using a GloMax-Multi Jr Single-Tube Multimode Reader (E6076, Promega) using the Cell-titre-Glo default settings.

### RNA extraction, quantitative PCR and RNASeq

RNA was extracted from cells using the miRNeasy miRNA isolation mini kit (#217004, Qiagen). To quantify mRNA of interest, cDNA synthesis and quantitative PCR (qPCR) were conducted using the GoTaq 2-Step RT-qPCR dye-based detection system (#A6010, Promega) and a Roche 480 Lifecycler (Roche, Sussex, UK). Quantification of microRNAs was achieved using the MystiCq microRNA cDNA synthesis mix and MystiCq microRNA qPCR dye-based assay primer system (#MIRRT, Sigma). Primers are detailed in Supplementary Table 3. For RNAseq samples were prepared using the Truseq small RNA library (#RS-200-0012, Illumina, Essex, UK), Truseq Stranded RNA library (#20020597, Illumina) and sequenced using the NextSeq 500 High Output Run (150 cycles) for two biological replicates. Two biological replicates were sequenced and analysis conducted using Cutadtapt, SHRiMP, SAMtools for trimming, mapping to GRch38 build, generating the raw counts. Data available from the Gene Expression Omnibus GSE117744. FunRich software was used to conduct enrichment analysis with Fishers exact test to generate *P* values [45].

### Protein extraction and immunoblots

Transfected cells were washed in PBS and lysed directly into 4 × Laemmli buffer (#1610747, Biorad, Watford, UK). Isolated EVs were lysed directly in 4 × Laemmli buffer (#1610747, Biorad). Immunoblots were performed as previously described [46]. All antibodies were purchased from Cell Signalling (NEB, Hitchin, UK) and were used at 1:1000 dilution: Dicer (D38E7, #5362 S), Beta-Tubulin (9F3, #2128 S), Annexin V (#8555 S), Alix (3A9, #2171 S), CD54/I-CAM (#4915 S), EpCAM (D1B3, #2626 S).

## Electronic supplementary material

Supplementary Legends

Supplementary Figure 1

Supplementary Figure 2

Supplementary Figure 3

Supplementary Figure 4

Supplementary Figure 5

Supplementary Figure 6

Supplementary Figure 7

Supplementary Tables 1 and 3

Supplementary Table 2
