# Supplementary material for: Communication of prostate cancer cells with bone cells via extracellular vesicle RNA; a potential mechanism of metastasis
Source: Oncogene. 2018 Oct 23;38(10):1751–63. doi: 10.1038/s41388-018-0540-5 (PMC6372071; doi:10.1038/s41388-018-0540-5)
Supplement: Supplementary file 1 — Supplementary Legends [file 41388_2018_540_MOESM1_ESM.docx]

**Supplementary Figure 1: Extracellular vesicle (EV) characterisation.**

Electrophoresis and Brownian motion video analysis combined with laser scattering microscopy were used to determine the **(a)** average concentration of EV particles isolated per assay setup **(b)** and the median size (peak) compared between cell lines. **(c)** Example traces and still frames of the video tracking analysis conducted per cell line. Traces were performed on 3802, 3733, 3689 and 3763 particles across in total for PNT1A, C4-2, PC3 and hOBs respectively. *ns = not significant, *** p<0.0001 (One-way Anova and Holm-Sidak correction)*

**Supplementary Figure 2: Expression of protein markers of extracellular vesicles.**

To further characterise the isolated EV particles, the protein content was probed by western blot to determine the expression of membrane markers CD9 (enriched in exosome fractions and GM130 (a non-vesicle negative control). Representative blot.

Supplementary Figure 3: **Extracellular vesicles isolated from prostate cancer cells with a bone metastatic propensity alter the cell viability of osteoblast (hFOb1.19).** Osteoblasts (hFOb1.19) were treated with extracellular vesicles (EVs) isolated from cultured prostate cancer cell lines PC3, C4-2, C42-B, PNT1A, the same hFOb1.19 cell line or no EV control, cell viability was measured after 24 hours using an MT luciferase assay. A significant increase in luciferase was detected in hFOb1.19 cells treated with EVs from prostate cancer cell lines PC3, C4-2 and C42-4B (p=0.032, p=0.0001, p=0.0001 respectively) (n=3).

**Supplementary Figure 4: Confirmation of Dicer knockdown and effects on the background expression of a panel of miRNAs in EV-treated osteoblasts.**

**(a)** Western blot confirmation of dicer knockdown in PC3 EV donor cells. **(b)** Due to the possibility of siRNAs being loaded into EVs, the average expression of six miRNAs were quantified in hOB cells treated with EVs isolated from PC3 cells treated with si-scr and si-dicer. There was no significant change in average miRNA expression, confirming no carry over effect of using si-dicer RNAi in the EV donating PC3 cells. (**c-d**) EV particles isolated following RNAi transfection were characterised by Electrophoresis and Brownian motion video analysis combined with laser scattering microscopy were used to determine the average concentration of EV particles per ml and the median vesicle size. Statistical significance was determined using an unpaired t-test with Welch’s correction.

** p=0.0456, ns = not significant.*

**Supplementary Figure 5: Validation of the presence of selected target genes in the EVs isolated from prostate cancer cell PC3 and C4-2.** RNA was extracted from EVs isolated form either the PC3 or C4-2 prostate cancer cell line. qPCR was used to determine the presence of each transcript used within the study to determine uptake of EV-mRNA by recipient osteoblast cells (Figure 4).

**Supplementary Figure 6: Detection of labelled mRNAs originating from bone-metastatic prostate cancer cell lines in recipient osteoblasts and the contribution to overall transcript abundance in recipient osteoblasts.**  Analysis of total RNA extracted from hOBs after treatment with labelled C4-2 EVs demonstrated similar results to those obtained when treating with PC3 EVs (Figure 5).

**Supplementary Figure 7: Comparison of the EV mRNA cargo with the predicted gene targets of the EV miRNA cargo.** RNAseq was used to identify the miRNA and mRNA molecules within PC3 vesicles. The predicted gene targets of the EV-miRNAs were generated and then compared to the known EV-mRNAs. Only 3.5% of the EV-mRNAs are targets of the EV-miRNAs. Target prediction and Venn diagrams were generated using FunRich [46].
