# Supplementary figures and images for: Communication of prostate cancer cells with bone cells via extracellular vesicle RNA; a potential mechanism of metastasis
Source: Oncogene. 2018 Oct 23;38(10):1751–63. doi: 10.1038/s41388-018-0540-5 (PMC6372071; doi:10.1038/s41388-018-0540-5)

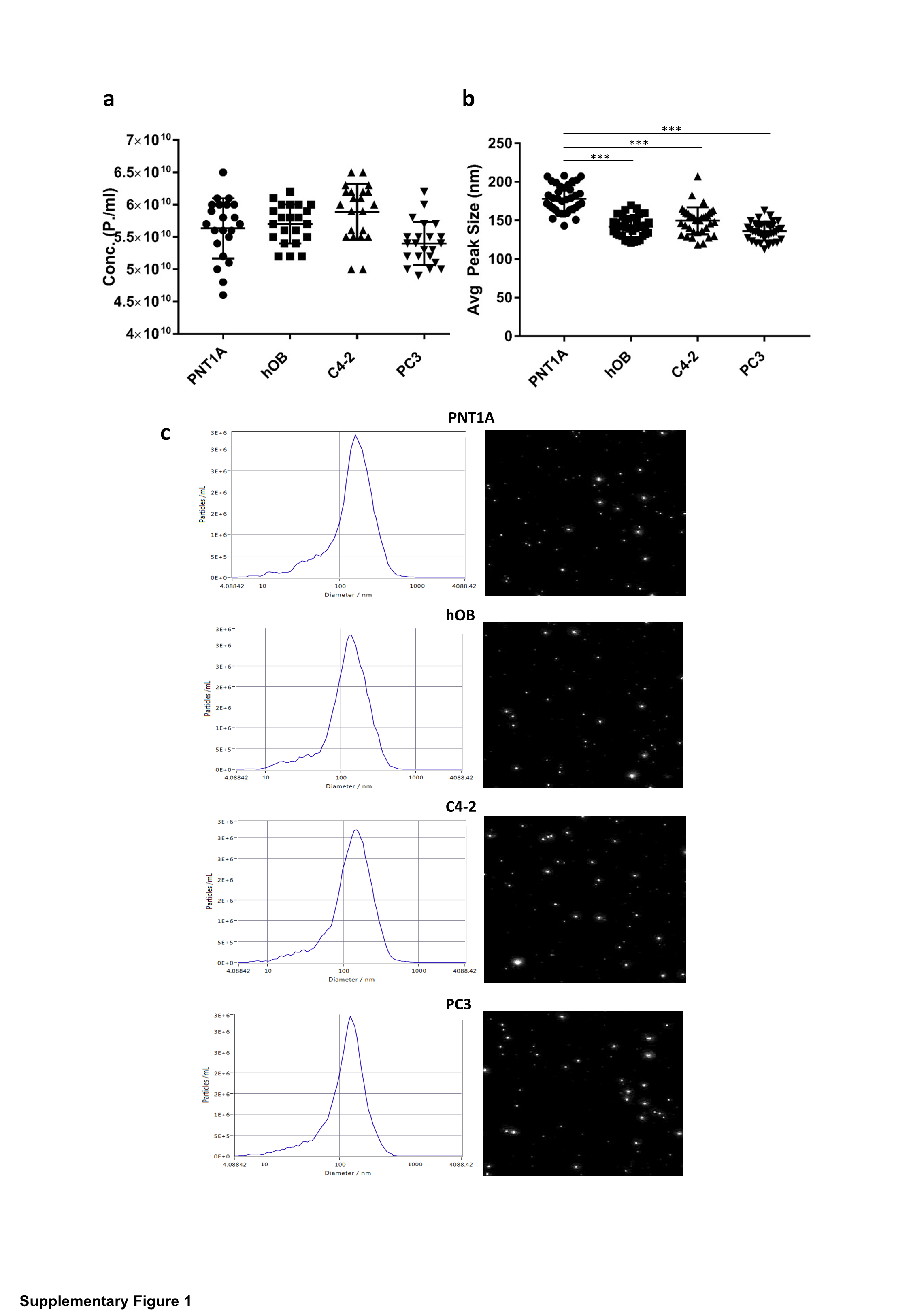

Supplement: Supplementary file 2 — Supplementary Figure 1 [file 41388_2018_540_MOESM2_ESM.tif]

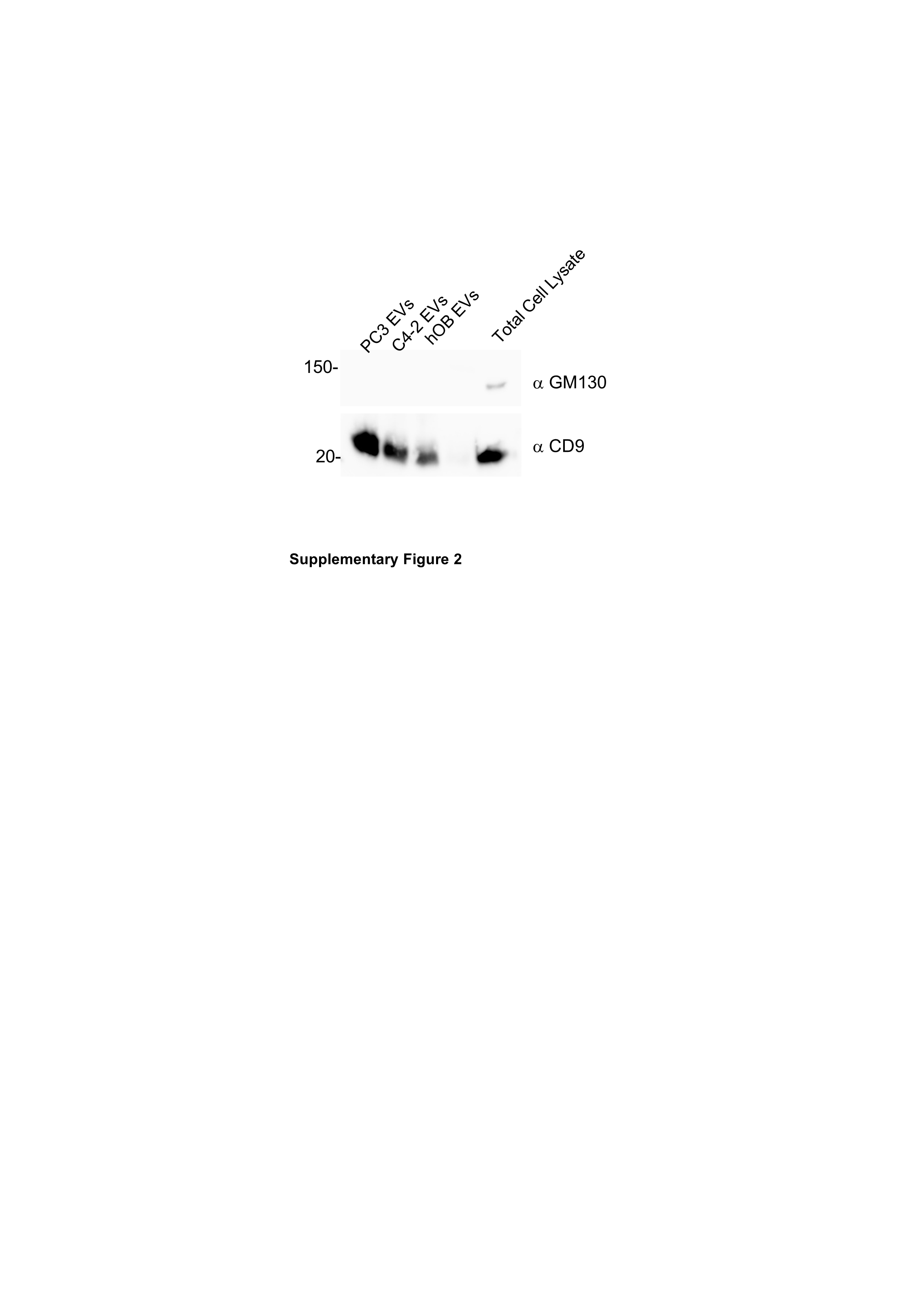

Supplement: Supplementary file 3 — Supplementary Figure 2 [file 41388_2018_540_MOESM3_ESM.tif]

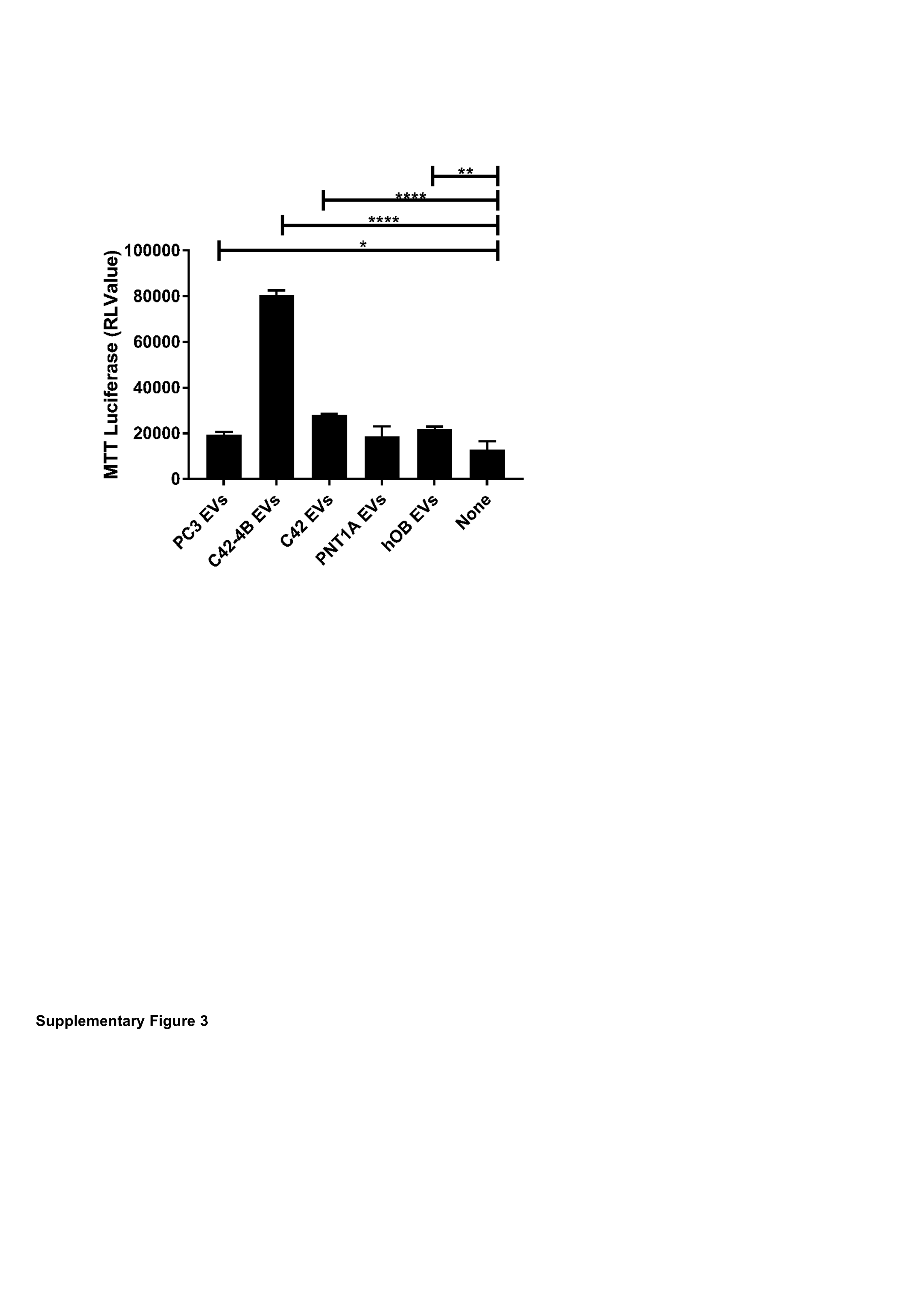

Supplement: Supplementary file 4 — Supplementary Figure 3 [file 41388_2018_540_MOESM4_ESM.tif]

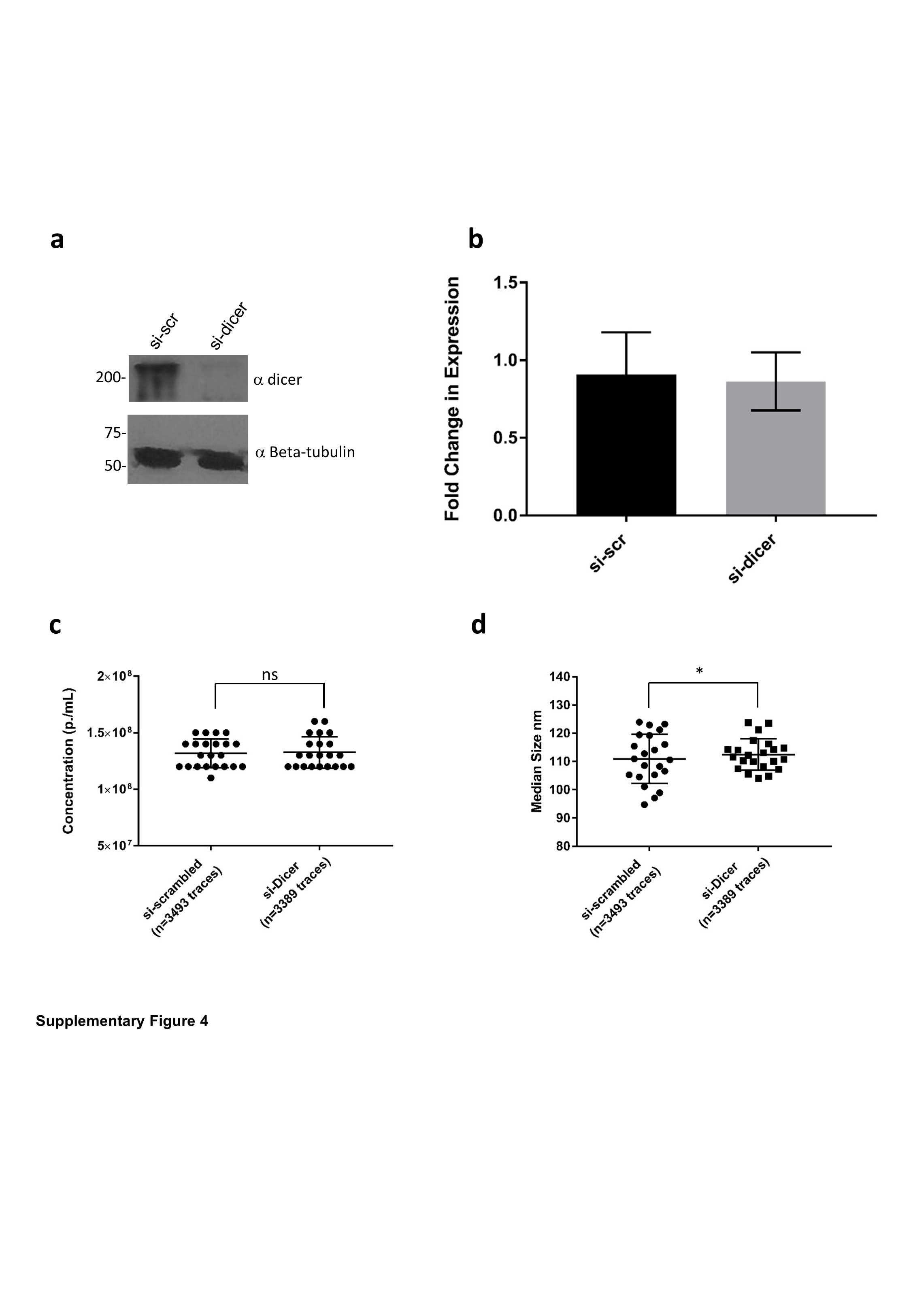

Supplement: Supplementary file 5 — Supplementary Figure 4 [file 41388_2018_540_MOESM5_ESM.tif]

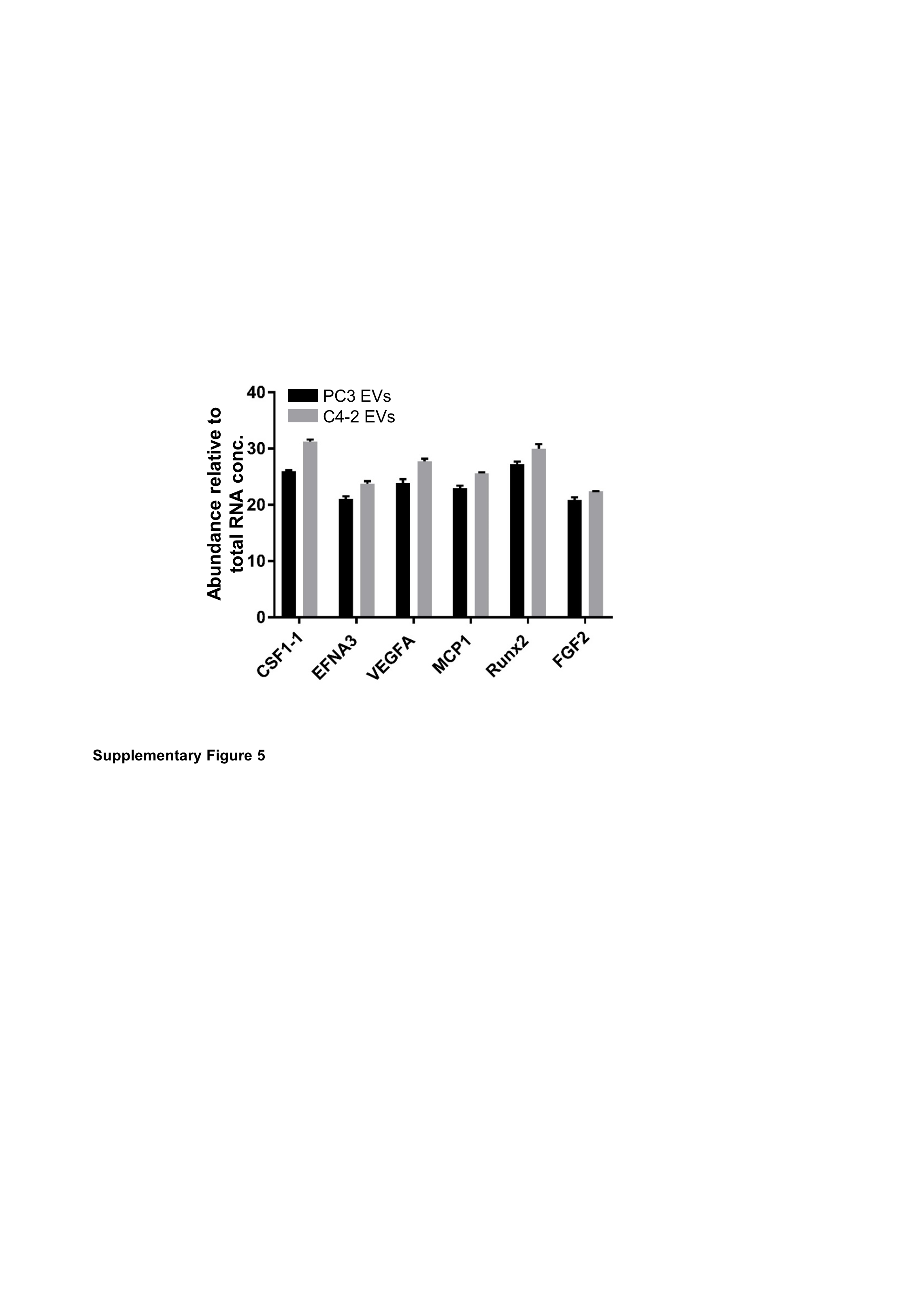

Supplement: Supplementary file 6 — Supplementary Figure 5 [file 41388_2018_540_MOESM6_ESM.tif]

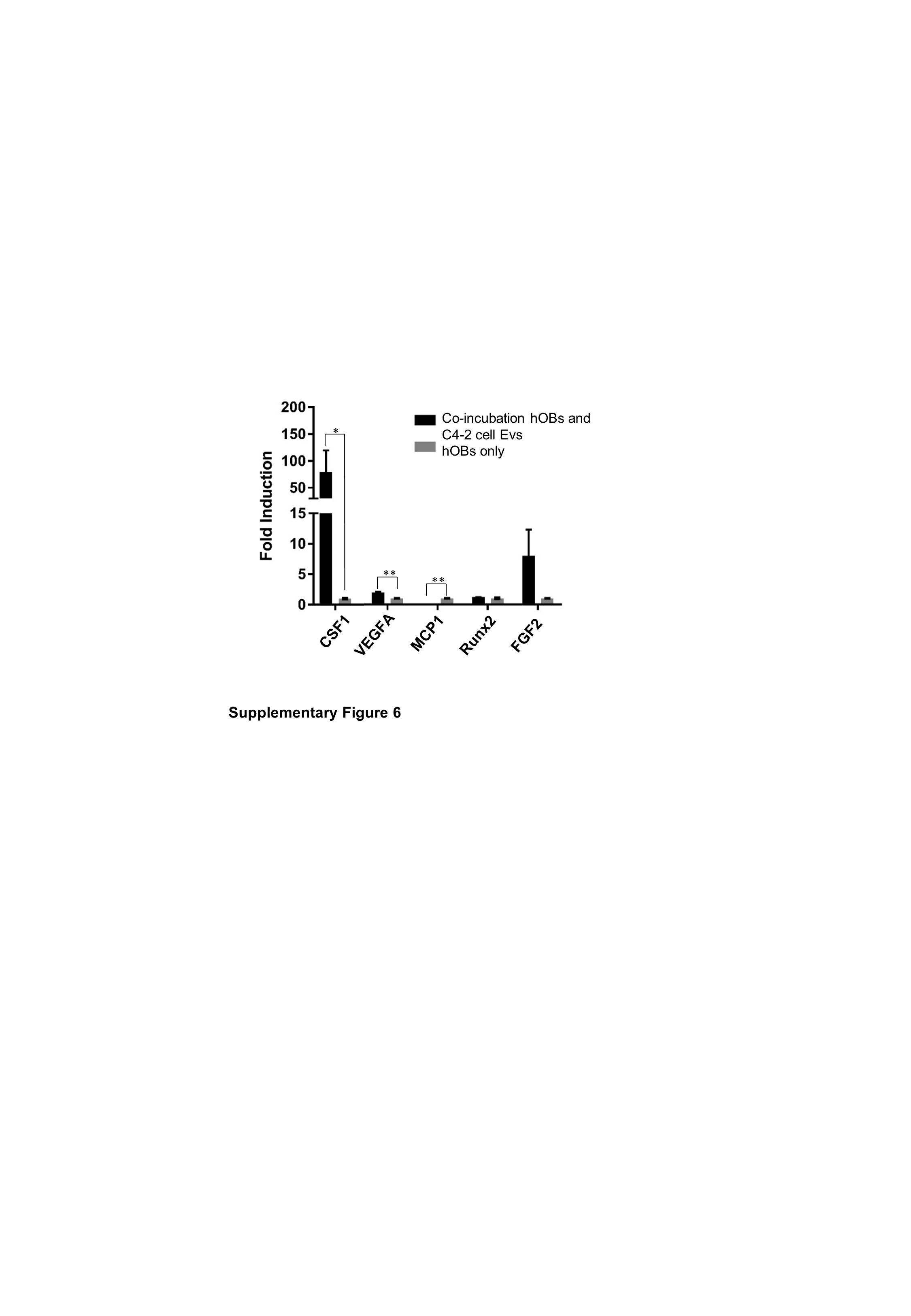

Supplement: Supplementary file 7 — Supplementary Figure 6 [file 41388_2018_540_MOESM7_ESM.tif]

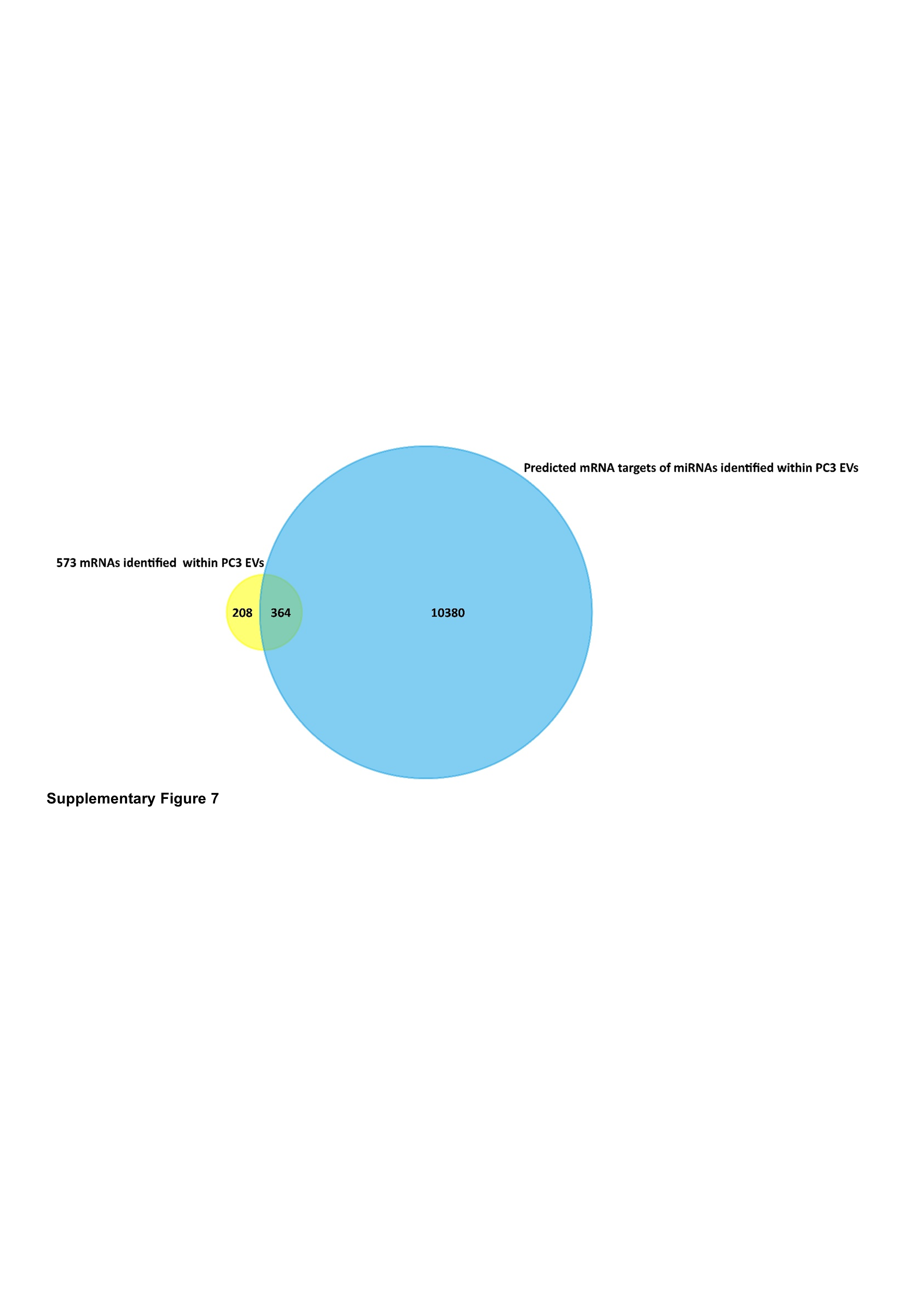

Supplement: Supplementary file 8 — Supplementary Figure 7 [file 41388_2018_540_MOESM8_ESM.tif]
