# Supplementary material for: Communication of prostate cancer cells with bone cells via extracellular vesicle RNA; a potential mechanism of metastasis
Source: Oncogene. 2018 Oct 23;38(10):1751–63. doi: 10.1038/s41388-018-0540-5 (PMC6372071; doi:10.1038/s41388-018-0540-5)
Supplement: Supplementary file 9 — Supplementary Tables 1 and 3 [file 41388_2018_540_MOESM9_ESM.docx]

| **miRNAs** | **Sample Type** | **Reference** |
| --- | --- | --- |
| *7 miRNAs shared amongst all compared studies (Probert 2017*; Hessvik 2012; Foj 2017; Rodriguez 2017; Bryant 2012, Huang 2015)* | | |
| Hsa-miR-574-3p | Plasma EV | Bryant, 2012 |
| Hsa-miR-501-3p | Urine EV | Rodriguez, 2017 |
| Hsa-miR-1290 | Plasma EV | Huang, 2015 |
| Hsa-miR-2110 | Plasma EV | Bryant, 2012 |
| Hsa-miR-107 | Plasma EV | Bryant, 2012 |
| Hsa-miR-331-3p | Plasma EV | Bryant, 2012 |
| Hsa-miR-375 | Plasma EV | Huang, 2015 |
|  |  |  |
| *6 additional miRNAs identified by comparing the Probert 2017* study of PC3 cell line EVs and EVs isolated from prostate cancer plasma and urine sample studies (Foj 2017; Rodriguez 2017; Bryant 2012, Huang 2015)* | | |
| Hsa-miR-21 | Plasma EV | Bryant, 2012 |
| Hsa-miR-625 | Plasma EV | Bryant, 2012 |
| Hsa-miR-301a | Plasma EV | Bryant, 2012 |
| Hsa-miR-143-3p | Urine EV | Rodriguez, 2017 |
| Hsa-miR-196a-5p | Urine EV | Rodriguez, 2017 |
| Hsa-miR-196a-3p | Urine EV | Rodriguez, 2017 |

**Supplementary Table 1: Details of microRNAs found in both our RNAseq analysis of EVs isolated from the prostate cancer PC3 cell line and those identified from EVs isolated from patient plasma and urine for which there is potential prognostic significance**

**Probert 2017 – identified in this study.*

**Supplementary Table 3: Details of the primers used within the study**

| **Gene Name** | **Forward Primer ^5’-3’^** | **Reverse Primer ^5’-3’^** |
| --- | --- | --- |
| CSF1 | GCGCTTCAGAGATAACACCC | TCTTGACCTTCTCCAGCAACT |
| RUNX2 | TGAACTCTGCACCAAGTCCT | GGGTGGTAGAGTGGATGGAC |
| MCP1 | GCAGCAAGTGTCCCAAAGAA | TCGGAGTTTGGGTTTGCTTG |
| VEGF-A | GCAGACCAAAGAAAGATAGACCAAG | CGCCTCGGCTTGTCACAT |
| EFNA3 | ACTCTCCCCCAGTTCACCA | GCACCTGAGGGTTCTCTCC |
| FGF2 | CCCTCACATCAAGCTACAACTTCA | TCCATCTTCCTTCATAGCCAGGT |
